# Enhanced Syllable Discrimination Thresholds in Musicians

**DOI:** 10.1371/journal.pone.0080546

**Published:** 2013-12-05

**Authors:** Jennifer Zuk, Ola Ozernov-Palchik, Heesoo Kim, Kala Lakshminarayanan, John D. E. Gabrieli, Paula Tallal, Nadine Gaab

**Affiliations:** 1 Laboratories of Cognitive Neuroscience, Developmental Medicine Center, Boston Children's Hospital, Boston, Massachusetts, United States of America; 2 Harvard Medical School, Boston, Massachusetts, United States of America; 3 Helen Wills Neuroscience Institute, University of California, Berkeley, California, United States of America; 4 Center for Molecular and Behavioral Neuroscience, Rutgers University, Newark, New Jersey, United States of America; 5 Department of Brain and Cognitive Sciences, Massachusetts Institute of Technology, Cambridge, Massachusetts, United States of America; 6 Harvard Graduate School of Education, Cambridge, Massachusetts, United States of America; Baycrest Hospital, Canada

## Abstract

Speech processing inherently relies on the perception of specific, rapidly changing spectral and temporal acoustic features. Advanced acoustic perception is also integral to musical expertise, and accordingly several studies have demonstrated a significant relationship between musical training and superior processing of various aspects of speech. Speech and music appear to overlap in spectral and temporal features; however, it remains unclear which of these acoustic features, crucial for speech processing, are most closely associated with musical training. The present study examined the perceptual acuity of musicians to the acoustic components of speech necessary for intra-phonemic discrimination of synthetic syllables. We compared musicians and non-musicians on discrimination thresholds of three synthetic speech syllable continua that varied in their spectral and temporal discrimination demands, specifically voice onset time (VOT) and amplitude envelope cues in the temporal domain. Musicians demonstrated superior discrimination only for syllables that required resolution of temporal cues. Furthermore, performance on the temporal syllable continua positively correlated with the length and intensity of musical training. These findings support one potential mechanism by which musical training may selectively enhance speech perception, namely by reinforcing temporal acuity and/or perception of amplitude rise time, and implications for the translation of musical training to long-term linguistic abilities.

## Introduction

Learning to play a musical instrument involves the development of a range of highly specialized perceptual, motor and cognitive skills (see [1] for an overview). Musicians learn to attend to the fine-grained acoustic properties of musical sound, read a symbolic system of musical notation, and translate these into highly coordinated and precise motor output. It has been suggested that intensive musical training is related to enhanced auditory processing abilities, such as heightened pitch discrimination [2], [3], [4], [5], [6], [7], harmonic sensitivity [8], [9], [10], [11], and differences in timbre [12]. Further, the degree of auditory enhancement observed in musicians has correlated with the length of musical study [8], 13,14. Musical ability has also been associated with superior perception of timing, such as rhythm and meter discrimination [15], [16], [17]. Thus, evidence has linked extensive musical training with enhanced perception of spectral and temporal elements of acoustic stimuli.

Speech and music overlap in spectral and temporal acoustic features and there is abundant evidence that musicians demonstrate enhanced speech perception and language abilities [13], [14], [18], [19], [20], [21], [22], [23], [24]. However, it remains unclear whether the auditory processing benefits afforded by musical training are all-encompassing or whether some acoustic cues, crucial for speech and language processing, may be selectively enhanced. Speech is inherently acoustic, produced through modification of the vocal tract and lips to filter the raw signal to a specific spectral fine structure (formants) that characterizes individual vowels and consonants [25]. These harmonic components are essential for speech discrimination, as is the fundamental frequency of the speaker's voice, which conveys the prosodic features of communication. Given that advanced acoustic perception is integral to accomplished musical skills, musical training may lead to benefits in processing these acoustic features on a fine-grained scale [26], [27], [28], [29], [30]. Further, musicians have demonstrated enhanced neural responses over non-musicians during sound discrimination in music and speech stimuli characterized by differences in frequency, duration and intensity [31]. The present study aimed to evaluate which acoustic features in speech, particularly in the spectral and temporal domains, may be superiorly processed by musicians over non-musicians.

Temporal features of speech, such as temporal aspects of segmental structure or the amplitude envelope of the signal, are another category of acoustic features that musical training has previously been associated with. Precise timing and synchrony are essential features that integrate musical segments, which are comparable to the specific temporal transitions within the formants that differentiate speech sounds in an utterance. Accordingly, research suggests that musical training enhances the perception of specific segmental (phonemic) components of speech. For instance, a positive relation between music perception abilities and phonological awareness, the ability to actively manipulate segmented speech sounds, has repeatedly been demonstrated [21], [22], [24], [32]. Furthermore, music-based interventions have led to improved performance in phonological processing and speech segmentation in typically developing school-age children as well as those struggling to read [19], [33], [34], [35], [36], [37], [38]. These auditory processing skills, as well as others such as non-linguistic auditory discrimination, have been suggested to be essential precursors to literacy (e.g. [39], [40], [41]). Thus, the importance of timing in segmentation of acoustic events is evident in both speech and music, with emerging, yet debated (e.g. [42]), evidence of direct linguistic benefit from musical training.

Additionally, the amplitude envelope conveys temporal information in speech (characterized by changes in the amplitude of a signal over time), and plays a significant role in speech segmentation [43], [44]. Consequently, slower amplitude modulations provided by the temporal envelope have been suggested as the most important acoustic cue for speech discrimination [45], [46], [47]. In music, the amplitude envelope has been implicated as an important characteristic of sounds, shown to be an essential feature of timbre [48] and a determining cue for distinguishing between musical instruments [49]. Thus, the amplitude envelope seems to play an important role in differentiation of acoustic information in both speech and music. Based on this evidence, the amplitude envelope has been proposed as critical to perception across both speech and music [29]. Therefore, it is intriguing to investigate whether musical expertise selectively enhances sensitivity to acoustic information provided by the temporal envelope, since this has yet to be explored in musicians versus non-musicians in speech-like stimuli.

Spectral features of speech, such as transitions in pitch throughout an utterance, are one category of acoustic features that musical training has previously been associated with. Specifically, superior perception of prosody, measured by matching natural speech utterances with the pitch-extracted equivalent of the same utterance, was enhanced in musically trained adults [50]. Musicians have further been shown to be better at detecting linguistic pitch manipulations in their native language, in a foreign language [51], [52], and in an artificial language comprised of pseudowords [53]. Thus, a number of studies propose that musical training selectively relates to superior perception of pitch in speech.

Since speech inherently encompasses both spectral and temporal properties, examining each of these attributes in isolation through synthesis at the *syllabic level* allows for observation of which of these acoustic properties in speech may be expertly processed by musicians. Several studies have done this, but findings are somewhat inconclusive. For instance, musical training has been associated with an increased subcortical sensitivity to spectral and temporal resolution of speech syllables [54], [55]. In contrast, enhanced cortical responses in musically trained children have been demonstrated for syllables that varied in temporal (through Voice Onset Time (VOT) and duration deviants) but not spectral domains, using electroencephalography (EEG) [30], [56]. However, behaviorally, musicians were more accurate and faster at discriminating speech sounds that varied in frequency as well as in temporal and VOT domains, thus revealing dissociation between neural and behavioral results [56]. The divergence between these behavioral, subcortical and cortical findings may be due to inconsistency among behavioral and neurophysiological responses, as has been previously found for differentiation of spectral acoustic features [57]. Nonetheless, it is apparent that the current evidence of which acoustic cues in syllabic speech may be superiorly processed by musicians remains inconclusive.

The present study aimed to evaluate the sensitivity of musicians to the acoustic components of speech necessary for intra-phonemic discrimination of synthetic syllables. To address the extent of the advantage for processing acoustic aspects of speech in expertly trained musicians, the present study investigated the discrimination threshold of three synthetic speech syllable continua that varied in their temporal and spectral discrimination demands, as well as VOT cues. While previous studies have described syllable processing in musicians through subcortical encoding [55] and acoustic manipulation of single-syllable pairs [56], this study aimed to identify which acoustic elements of speech are most closely associated with musical ability through a task paradigm that evaluates the threshold of discrimination across three detailed continua. The three speech syllable continua implemented in this study each isolate specific acoustic features characteristic of speech at the syllabic level as follows: /ba/-/da/ (spectral change within the formant transition), /ba/-/wa/ (spectral and temporal change within the formant transition), and /ga/-/ka/ (change in VOT). Since musical training has been previously associated with sensitivity to segmental distinctions in speech [5], [18], [30], [37], [56], we predict that musicians will perform better on syllable continua that capture temporal changes (/ba/-/wa/ and /ga/-/ka/). In addition, amplitude envelope will be evaluated across all syllable continua to investigate the relation between any group effects and temporal changes revealed by the envelope. As for spectral sensitivity, there are inconsistencies in the current literature regarding whether musicians demonstrate enhanced perception of spectral aspects of speech, especially on the syllabic level. Thus, we will further explore this question by evaluating discrimination thresholds in musicians versus non-musicians on the continuum characterized by mainly spectral changes (/ba/-/da/). Overall, the present study aimed to investigate the acoustic sensitivity of musicians across several features within the speech domain through a task that offers a more fine-grained analysis of syllable discrimination than has been utilized to date.

## Methods

### Participants

28 young adults (14 male, 14 female, ages: 18–25, mean = 19, SD = 1) were recruited from Stanford University. The group included 14 musicians with a minimum of nine years of experience (mean = 13, SD = 3) playing a musical instrument and who practiced a minimum of five hours per week (mean = 10, SD = 4) for the last five years. There were seven females in each group. Three musicians were instructed in string instruments, five in piano, and six in woodwind instruments. Non-musicians had less than three years (mean = 2, SD = 2) of instrumental experience, and no musical involvement within five years of study participation. All participants were right handed, native speakers of American English, had no background of a tonal language, and reported to not have absolute pitch or have ever had a history of language, reading or learning disability. One musician reported to have a brother who stuttered as a child, one musician reported a family member with undiagnosed dyslexia, and two non-musicians reported having a family member with a diagnosis of developmental dyslexia. The study was approved by Stanford's Administrative Panels for the Protection of Human Subjects. All participants provided informed written consent and were compensated for their participation.

The groups showed no significant difference for age, gender, intellectual ability (Shipley Institute of Living Scale; SILS [58]), or reading abilities (Word Identification and Word Attack subtests of the Woodcock Reading Mastery Test-Revised [59]), [Table 1]. The SILS consists of two subtests: Vocabulary and Abstraction. The Vocabulary subtest consists of 40 multiple-choice questions in which the respondent is asked to choose which of four words is closest in meaning to a target word. The Abstraction subtest consists of 20 questions in which sequences of numbers, letters or words are presented with the final element in each sequence omitted. The respondent is required to complete each of the sequences. The Word Identification subtest of the Woodcock Reading Mastery Test-Revised requires participants to read isolated words aloud. The Word Attack subtest requires participants to read either nonsense words or words with a very low frequency of occurrence in English; it measures the ability to apply phonic and structural analysis skills to pronounce unfamiliar words. This measure was applied in order to measure single word reading abilities and in order to rule out current reading impairments.

**Table 1:** Characteristics of musicians and non-musicians.

	Musicians	Non-Musicians
Age in Years	19.7 (0.40)	18.9 (0.26)
Years Played***	12.6 (0.79)	1.5 (0.42)
Hours Played/Week***	10.3 (1.06)	0 (0.0)
Word ID (standard score)	118.14 (1.84)	115.14 (1.65)
Word attack (standard score)	119.79 (3.04)	121.29 (2.65)
IQ (verbal and abstract)	122.286 (3.22)	121.64 (3.83)

*Standard errors are reported in parentheses*.

*** *p<0.001*.

### Stimuli

Three different synthetic speech syllable continua were implemented in this experiment: /ba/-/da/ (spectral change within formant transition), /ba/-/wa/ (duration change of formant transition), and /ga/-/ka/ (change in Voice Onset Time). The /ba/-/da/ contrast was derived by manipulating the onset of the second formant, primarily a spectral change, while retaining the duration of the formant transition (equally brief at 40 ms) for all stimuli in the continuum. The /ba/-/wa/ continuum was created by manipulating the duration of the formant transition from 25 to 100 ms, while the /ga/-/ka/ continuum was created by manipulating the Voice Onset Time (VOT); both primarily involve a temporal change. The acoustic parameters used to synthesize the three continua are specified below. All syllables were 200 ms in duration and were produced using a Klatt-based synthesizer [60]. All syllables had a fundamental frequency (F0) of 120 Hz, which dropped to 90 Hz through the duration of the syllable. The synthesizer used for this study limited the resolution of the step size used in each syllable continuum.

**Figure 1:**
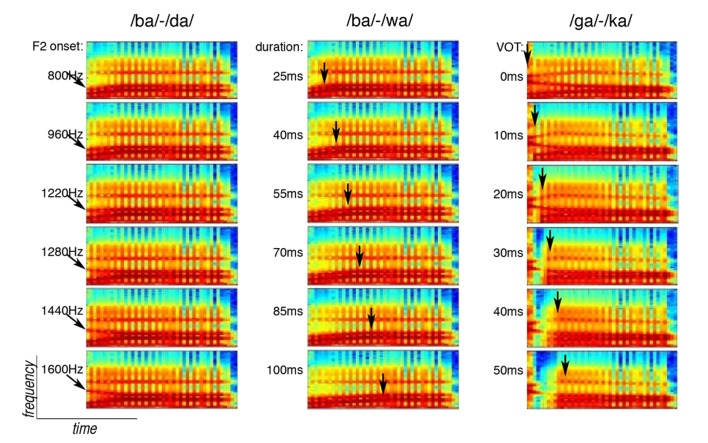
Experimental Stimuli: The spectrograms show the end points of the three continua (/ba/-/da/, /ba/-/wa/, /ga/-/ka/) and four intermediate points. The onset value of the second formant in the /ba/-/da/ continuum varied from 800–1600 Hz (see black arrows). The duration of the formant transition in the /ba/-/wa/ continuum varied from 25–100 ms (black arrows). The Voice Onset Time (VOT) of the first formant in the /ga/-/ka/ continuum varied from 0–50 ms (black arrows).

/ba/-/da/ continuum (Figure 1): The onset value of the second formant for the /ba/-/da/ continuum varied from 800 to 1600 Hz, (/ba/ and /da/, respectively), in 40 Hz steps producing 21 syllables spanning a spectral continuum between /ba/ and /da/. The starting frequencies for the formant transitions of the /ba/-/da/ continuum were: F1 = 420 Hz, F2: varying from 800 to 1600 Hz, F3 = 500 Hz, F4 = 3250 Hz, and F5 = 3700 Hz. The transition was 40 ms, at which point the formant frequency (F) and bandwidth (BW) values were: F1 = 800 Hz, BW1 = 90; F2 = 1200 Hz, BW2 = 110; F3 = 2500 Hz, BW3 = 90; F4 = 3250 Hz, BW4 = 400; F5 = 3700 Hz, BW5 = 500. At 180 ms, the formant frequency changes were: F1 = 750 Hz and the voicing was ramped down to zero for the remaining 20 ms.

/ba/-/wa/ continuum (Figure 1): The duration of the transition varied from 25 to 100 ms, (/ba/ and /wa/, respectively), in 5 ms steps, producing 16 syllables along this continuum. The frequency and bandwidth specifications were identical to the /ba/ used in the /ba/-/da/ continuum (see above) except F2 remained 800 Hz and the transition duration varied from 25 to 100 ms.

/ga/-/ka/ continuum (Figure 1): The Voice Onset Times (VOTs) for the /ga/-/ka/ spectrum ranged from 0 to 50 ms, (/ga/ and /ka/, respectively) in 5 ms steps producing 11 syllables along this continuum. The starting frequencies for the formant transitions were: F1 = 420 Hz, F2 = 1625 Hz, F3 = 2125 Hz, F4 = 3250 Hz, and F5 = 3700 Hz. The formant frequency and bandwidth values at the beginning of the vowel were: F1 = 700 Hz, BW1 = 90; F2 = 1200 Hz, BW2 = 90; F3 = 2600 Hz, BW3 = 130; F4 = 3250 Hz, BW4 = 400; F5 = 3700 Hz, BW5 = 500. At 180 ms, the formant frequency changes were: F1 = 750 Hz, F2 = 1000 Hz, F3 = 2300 Hz and the voicing was ramped down to zero for the remaining 20 ms.

### Procedure

Participants were seated comfortably in a quiet testing room with a PC computer running ePrime [61]. Stimuli were transmitted to participants through Panasonic stereo headphones. The sound intensity was adjusted according to each participant's preferred level to ensure optimal perception of the stimuli. Participants heard a pair of syllables, one presented after the other with an inter-stimulus interval of 750 ms, and had to respond whether the two syllables were the same or different using buttons on a keyboard. Each pair contained a fixed reference syllable (/ba/, /ba/, or /ga/ depending on the continuum) and a test syllable. During presentation the order of reference and test syllable was randomized. Trial progression was according to three-down one-up adaptive staircase method [62]. At the beginning of each assessment, the test syllable was at the opposite end of the continuum from the reference syllable; that is, trials always began with the most easily discriminable stimulus pair from the continuum. For the /ba/-/da/ continuum, the discrimination limen of the first stimulus pair corresponded to 800 Hz; thus, the second formant frequency at each syllable onset, /da/ at 1600 Hz and /ba/ at 800 Hz, marks the extremes of the continuum. Using the same approach, the discrimination limen of the first pair for the /ba/-/wa/ continuum would be 75 ms and for the /ka/-/ga/ continuum it would be 50 ms. After three consecutive correct responses to the first pair, the discrimination limen was decreased by two steps and the trial progressed to the next levels of difficulty accordingly. For each incorrect response, the discrimination limen was increased by one step and an easier stimulus pair in the continuum was presented until 7 reversals in the direction of progression of trials were achieved. Catch trials containing pairs of identical syllables were presented every 5–10 trials (for which all participants performed at 100%). Each assessment was terminated after 7 reversals or 5 consecutive incorrect responses to the initial, most easily distinguished pair. The discrimination thresholds for each of the stimulus continua were determined by the arithmetic mean of the discrimination limen corresponding to the last 4 reversals. The original threshold value was measured in Hz for /ba/-/da/ and in ms for /ba/-/wa/ and /ga/-/ka/.

In order to allow direct comparison between the three syllable continua, the discrimination thresholds in Hz and ms were transformed into a Relative Threshold Index (RTI) ranging from 0 to 1. Specifically, the RTI was the value obtained by subtracting the reference syllable value (for /ba/-/da/ 800 Hz, for /ba/-/wa/ 25 ms and for /ga/-/ka/ 0 ms) from the obtained discrimination threshold. This number was then divided by the maximum range for each acoustic continuum (for /ba/-/da/ 800 Hz, for /ba/-/wa/ 75 ms and for /ga/-/ka/ 50 ms) and subtracted from 1 (see Table 2). Thus, a higher RTI indicates better discrimination. For instance, a discrimination threshold of 1400 Hz for the /ba/-/da/ continuum will have an RTI of 0.25, while a discrimination threshold of 1000 Hz will have an RTI of 0.75.

**Table 2:** Characteristics of the Syllable Pairs/Calculation of Relative Threshold Index.

Syllable Pair	Reference Syllable	Original Threshold	Relative Threshold Index
/ba/-/da/800 Hz–1600 Hz	/ba/ (800 Hz)	*x* Hz	1- [*x* Hz - 800/(1600 Hz–800 Hz)]
/ba/-/wa/25 ms–100 ms	/ba/ (25 ms)	*x* ms	1- [*x* ms - 25/(100 ms–25 ms)]
/ga/-/ka/0 ms–50 ms	/ga/ (0 ms)	*x* ms	1- [*x* ms/(50 ms–0 ms)]

Before each assessment, participants completed a practice run of 5 syllable pairs to familiarize themselves with the stimuli. The presentation order of the three continua was counterbalanced between participants. Two-sample t-tests between the two groups as well as repeated measurement ANOVAs and post-hoc tests within groups were calculated. Significance thresholds were corrected for multiple comparisons by controlling for the false discovery rate (FDR). The /ba/-/da/ and /ba/-/wa/ continua were further evaluated through envelope extraction and amplitude rise time analysis, as previously described by Nittrouer and colleagues [63]. Amplitude rise time was determined through identification of the amplitude peak of the syllable, followed by calculation of the root-mean-square (RMS) amplitude over the five pitch periods with the amplitude peak at the center, using WavEd software [64]. RMS amplitude was determined for individual pitch periods preceding the amplitude peak, and the first pitch period with an RMS value ≥80% of the peak amplitude value was defined as the end of the rise time. Amplitude rise time was then reported as the duration between the onset of the syllable and the end of the amplitude rise. Envelope and rise time findings were then replicated with Spike2 software (http://www.ced.co.uk/pru.shtml?spk7wglu.htm). To investigate the relationship between the intensity of musical practice and the discrimination threshold for each syllable continuum, Pearson correlations were performed between RTI and the number of years of musical practice, as well as the average weekly hours of musical practice during the time of the study.

## Results

**Figure 2:**
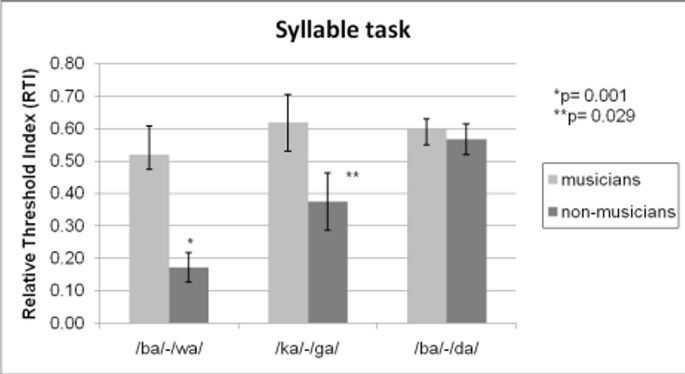
Result for the syllable discrimination task: Error bars indicate standard errors of the means. Higher relative threshold index scores indicate better discrimination abilities. The /ba/-/wa/ and /ga/-/ka/ continua are differentiated by a temporal change and the /ba/-/da/ continua is differentiated by a spectral change. Musicians showed significantly higher discrimination thresholds in the two continua differentiated by a temporal change (/ba/-/wa/ and /ga/-ka/).

The relative threshold indices (RTI) for the three syllable continua were analyzed in a 2×3 ANOVA with musicianship as a between-subjects factor and syllable continua relative threshold indices as repeated measures. Musicians performed better than non-musicians (*F*(1, 26) = 9.38, *p*<0.005, corrected for multiple comparisons according to the False Discovery Rate (FDR) criterion [65]), some syllable continua were more difficult than other continua (*F*(2, 52) = 7.68, *p*<0.001, FDR corrected), and there was a significant interaction between groups and continua (*F*(2, 52) = 3.46, *p*<0.05, FDR corrected). The interaction was examined via one-tailed two-sample t-tests due to our a priori hypotheses. Musicians had significantly better relative threshold indices than non-musicians for the two continua that differed on temporal information, the /ba/-/wa/ (*t*(26) = 3.44, *p*<0.001, FDR corrected) and /ga/-/ka/ continua (*t*(26) = 1.97, *p*<0.05, FDR corrected), but there was no significant difference between the groups for the /ba/-/da/ continuum that differed on spectral information (*t*(26) = 0.55, *p* = 0.29, FDR corrected), as outlined in Figure 2.

The main effect for the syllable continua was further examined using paired t-tests. Lower relative threshold indices were observed for the syllable continua /ba/-/wa/ compared to /ga/-/ka/ (*t*(27) = 2.38, *p*<0.05, FDR corrected) and /ba/-/wa/ compared to /ba/-/da/ (*t*(27) = 3.47, *p*<0.0005, FDR corrected). Correlation analysis revealed that total years of musical practice was positively related to threshold indices for the /ba/-/wa/ continua (r = 0.50, p<0.005) and a trend was observed for the /ga/-/ka/ continua (r = 0.28, p<0.1). Performance on /ba/-/da/ was not found to correlate significantly with length of musical practice. Additionally, the reported numbers of hours spent playing an instrument per week at the time of participation positively correlated with performance on the /ba/-/wa/ (r = 0.47, p<0.01) and /ga/-/ka/ (r = 0.35, p<0.05) continua but not on the /ba/-/da/ continua. No effects were observed between the type of musical instrument played and syllable discrimination performance.

As for envelope extraction analysis, amplitude rise time was found to be consistent for all syllables along the /ba/-/da/ continuum. By contrast, the /ba/-/wa/ continuum resulted in changes in the amplitude rise time duration across the syllables. Specifically, the amplitude rise time of /ba/ was found to be shorter than that of /wa/ on either end of the continuum. The /ga/-/ka/ envelope analysis was not reported, since the envelope identically represents the VOT. Thus, musicians demonstrated enhanced sensitivity to the two continua characterized by changes in the amplitude envelope (/ba/-/wa/) and the timing of acoustic onsets (/ga/-/ka/).

## Discussion

Musical training was associated with superior processing of temporal components of synthesized speech syllabi, as represented by amplitude rise time and VOT. Musicians demonstrated better discrimination thresholds relative to non-musicians for continua requiring intra-syllabic temporal resolution of acoustic cues (/ba/-/wa/ and /ga/-/ka/), but not for continua that differed in primarily spectral information (/ba/-/da/). Amplitude envelope extraction confirmed that the /ba/-/wa/ continuum varied in amplitude rise time, indicating that musicians in our sample showed increased sensitivity over non-musicians to discrimination of stimuli differentiated by amplitude envelope cues. Furthermore, the length and intensity of musical training positively related to performance on the /ba/-/wa/ and /ga/-/ka/ continua but not the /ba/-/da/ continuum. Thus these results suggest that musical training is selectively associated with enhanced perception of temporal features at the syllabic level.

These findings contribute to prior evidence seeking to identify the acoustic features of speech that may be processed by musically trained individuals with enhanced sensitivity. Consistent with our results, previous studies have identified enhanced perception of specific segmental components of speech following musical training in school-aged children [18], [30], [37]. Demonstrating neurobiological evidence for this advantage, others have shown that adults and children with musical training exhibit superior subcortical encoding of speech syllables [54], [55] and that children have heightened cortical responses to duration and VOT deviants in syllabic stimuli directly following controlled musical training [30]. Thus, our findings that musicians are more sensitive than non-musicians to the segmental and temporal transitions in speech sounds are in line with the previous literature. Our novel behavioral paradigm, measuring the intra-phonemic discrimination threshold of syllables for these acoustic parameters, and directly manipulating them through speech synthesis, has further demonstrated this specialization in musicians for temporal processing in speech.

Our results of the amplitude envelope extraction indicate that musicians in our sample showed lower thresholds over non-musicians in discrimination of stimuli differentiated by amplitude envelope cues. Studies investigating the acoustic components that are most essential for speech additionally suggest that slowly varying amplitude envelope information may be most important for speech discrimination [45], [46]. Studies in individuals with language-based impairments (e.g. specific language impairment and dyslexia) provide further insight on the acoustic cues necessary for higher-order linguistic abilities, as it has been suggested that these individuals exhibit poor sensitivity to various components of music [66], [67], [68] and are less sensitive in processing auditory temporal features (e.g. [40], [69], [70], [71], [72], [73]). These deficits have been linked with failure to establish a robust phonological schema of speech sounds, which in turn has been linked to difficulties in language processing and reading [39], [74]. Accordingly, amplitude rise time has since been proposed to be pivotal for representation of phonological structure and facilitation of speech segmentation [34], [75]. Our results suggest that musical training is specifically related to perception of amplitude rise time and VOT at the syllabic level, supporting previous hypotheses that the perception of temporal distinctions is integral to both musical training and speech perception [26], [76].

The present findings did not reveal enhanced perception of spectral changes in syllables in musicians over non-musicians. Previously, it has been suggested that musicians show enhanced perception of spectral features in linguistic phrases, such as pitch incongruences and/or prosody across a sentence [2], [50], [53]. These studies encompass a larger scope and require the evaluation of different spectral attributes compared to those examined in the present study. At the syllabic level, inconsistencies present in the current literature as to whether musicians demonstrate enhanced perception of spectral aspects of speech [30], [55], [56]. One prior study in musicians that implemented intra-syllabic stimuli did demonstrate enhanced subcortical distinction of syllables that varied in their second formant frequency, suggesting a neural advantage for encoding spectral information over non-musicians [55]. The divergence between our behavioral results and these subcortical findings may be due to inconsistency among behavioral and neurophysiological responses, as has been previously found, for differentiation of spectral acoustic features [57]. A behavioral advantage has also been previously reported in musicians for identifying the deviant syllable based on spectral changes [56]. However, the present syllable continua threshold of discrimination may be more sensitive than the previously employed oddball paradigm. Therefore, it is possible that the acoustic training specific to music does not enhance spectral processing in the speech domain. This is conceivable, since musical performance involves careful attention to the precise spectral and temporal transitions within musical phrases and/or the harmonic structure created by several instruments in an ensemble, rarely relying on spectral changes in isolation. Accordingly, 12 months of musical but not painting training in young children resulted in enhanced cortical responses to syllabic duration and VOT, as demonstrated by greater MMN amplitude through ERP recordings, but no changes in frequency processing were seen [30]. In addition, phonological decoding has been linked to subcortical timing and encoding of harmonic properties of speech, but not to pitch encoding [77]. Therefore, our data contribute to a growing body of evidence suggesting that musical training does not correlate with superior perception of spectral transitions in speech.

Our data should be interpreted in the context of several considerations. Firstly, the present study is unable to address the potential contribution of working memory or attention since no specific measures of these constructs were acquired. Another consideration is that although musicians and non-musicians did not differ in general language ability in the presently administered psychometric battery, the measures included here may not be sensitive to the advantages that musical training might demonstrate, such as second language acquisition [78]. It is also important to note that syllables in our study were presented along continua demanding comparative discrimination, whereas syllables in natural speech would be perceived categorically. Nonetheless, it is possible that participants relied more heavily on internal categorical templates for each syllable rather than direct sensitivity to acoustic manipulations along the continua, which may have influenced determination of the discrimination limen. Lastly, the implementation of synthesis tools with more naturalistic stimuli, such as MBROLA [79] or interpolation of naturalistic speech, would be advantageous in future investigations to determine whether these effects hold true for naturalistic speech that is manipulated along comparable continua.

The nature of this study precludes determining whether the differences in synthesized speech discrimination exhibited by musicians versus non-musicians are a consequence of musical training, or rather a predisposition to train in music. This question was partially addressed in this study by demonstrating the positive relationship between the length of musical practice and intensity and superior performance on temporal syllable continua. This finding suggests that the degree of perceptual sensitivity is commensurate with amount of musical experience; however, the role of innate predisposition cannot be ruled out. Furthermore, the small sample size in our study prevented us from evaluating potential differences in syllable perception based on the various types of instruments studied. It is possible that a larger sample of musicians studying instruments that demand fine-grained pitch discrimination may reveal an interaction between training and spectral discrimination in speech. Therefore, future empirical work is needed to determine whether specific acoustic attributes shared by speech and music are influenced by type of training, for example, if string instrumentalists are specialized in the pitch domain as has been previously suggested [7], if percussion instrumentalists are attuned specifically to timing and rhythm [76], or if individuals with extensive vocal training show enhanced speech perception.

Overall, this study demonstrates that musical training is associated with heightened sensitivity to temporal acoustic cues, as represented by amplitude rise time and VOT, of digitized speech syllables. These findings provide implications for the potential of music-based interventions in benefiting individuals with various auditory processing deficits. In particular, music-based interventions that emphasize temporal structure may enhance sensitivity to amplitude rise time and, in turn, promote improved language-processing abilities. Accordingly, several studies have already revealed promising results on the effects of rhythm-based music instruction on phonemic awareness [34], [35], [38], [80]; however, the long-term translation to improved language and reading abilities has yet to be established. Additional longitudinal intervention studies are needed to replicate these findings with a larger sample to determine the direct relation of musical training to temporal acoustic features of speech and potentially long-term linguistic abilities.
